# Cardiovascular events associated with thalidomide and prednisone in leprosy type 2 reaction^☆^

**DOI:** 10.1016/j.abd.2023.01.006

**Published:** 2023-08-30

**Authors:** Melissa de Almeida Corrêa Alfredo, Juliano Vilaverde Schmitt, Anna Carolina Miola, Simone de Pádua Milagres, Joel Carlos Lastoria

**Affiliations:** Department of Infectology, Dermatology, Diagnostic Imaging and Radiotherapy, Faculty of Medicine, Universidade Estadual Paulista, Botucatu, SP, Brazil

**Keywords:** Leprosy, Thalidomide, Venous thrombosis

## Abstract

**Background:**

Thalidomide is the drug of choice for the treatment of type 2 leprosy reactions and is often associated with corticosteroids. The use of these drugs in multiple myeloma is associated with the risk of cardiovascular events, but there have been few studies assessing this risk in leprosy patients.

**Objective:**

To evaluate the occurrence of cardiovascular events in patients with multibacillary leprosy and their correlation with the use of thalidomide and prednisone.

**Methods:**

Analytical cross-sectional study of all patients diagnosed with multibacillary leprosy treated at the Dermatology Service between 2012 and 2022, using electronic medical records. Thromboembolic vascular events, both arterial and venous, including acute myocardial infarction, were considered. The main independent variable was the concomitant use of thalidomide and prednisone during follow-up.

**Results:**

A total of 89 patients were included, of which 19 used thalidomide and prednisone concomitantly. There were five cardiovascular events (26.3%), three of which of deep venous thrombosis. The combined use of medications was associated with the events (PR = 6.46 [3.92 to 10.65]; p < 0.01).

**Study limitations:**

Small number of events, single-center retrospective study.

**Conclusion:**

The hypothesis of an association between cardiovascular events and the concomitant use of thalidomide and prednisone is supported, but more robust prospective studies are required for a better assessment.

## Introduction

Leprosy is an infectious and contagious disease caused by *Mycobacterium leprae*, which has a chronic evolution often interrupted by periods of symptoms exacerbation, called leprosy reactions. The disease has a varied spectrum of clinical manifestations depending on the host predominant immune response pattern against the bacillus. Type 2 leprosy reactions, or erythema nodosum leprosum, occur in patients with the multibacillary form of the disease and may persist or recur for several years after the end of the antimicrobial treatment.^1^, ^2^

Currently, thalidomide is one of the most commonly used drugs for this condition, as it is effective and has fewer adverse effects during long-term use when compared to corticosteroids or other immunosuppressants.^3^ On the other hand, the use of this medication and similar ones in the treatment of multiple myeloma, especially when associated with corticosteroids and chemotherapy, has shown an increased risk for thromboembolic vascular events.^4^

Until recently, this risk was attributed to the underlying hematological disease; on the other hand, mainly in the last two decades, several reports of thromboembolic events in patients using thalidomide and corticosteroids together in conditions other than multiple myeloma have been published, with several cases highlighted in leprosy patients.^5^, ^6^

Recently, a cross-sectional study carried out in the state of Minas Gerais showed the prevalence of deep vein thrombosis (DVT) in 10% of cases of adverse effects to thalidomide. Of the ten patients who experienced the thromboembolic effect, nine were on concomitant prednisone.^7^

Aiming to explore this association, an analytical cross-sectional study was carried out, including all patients with multibacillary leprosy attended at the Dermatology Service of Hospital das Clínicas, Faculty of Medicine, in Botucatu - UNESP and followed for at least four months, since leprosy type 2 reactions and the use of thalidomide usually begins after starting multidrug therapy and the thrombotic phenomena were reported as occurring on average 45 to 90 days after the use of the medication in cancer patients.^8^

## Methods

Data were obtained between February 2020 and January 2022 by searching the electronic medical records of patients attended at the Leprosy Outpatient Clinic of the Dermatology Service between 2012 and 2021, after approval by the Research Ethics Committee (CAAE: 57896422.6 .0000.5411/Counsel n. 5,410,411). Patients with a short time of follow-up (< four months) and paucibacillary disease, as well as those with no established diagnosis of leprosy, were excluded. Both arterial and venous cardiovascular events (CVEs) were considered. The time of follow-up was defined as the end of the dermatological follow-up with outpatient discharge, or until the occurrence of a thromboembolic event. Collected data included sex, age at the start of the treatment, time of follow-up, occurrence of an event, type of event, use of thalidomide during follow-up associated or not with corticosteroids, report of systemic arterial hypertension (SAH), diabetes mellitus (DM) or smoking during follow-up.

## Results

The study included 89 multibacillary patients, with a mean age of 55 (±14) years, 68.5% men, followed for a median period of 39 (1^st^ q-3^rd^ q: 19‒70) months. A total of 22.5% of the patients were smokers, with no association with gender (p = 0.59). During the study period, 19 patients had active erythema nodosum leprosum and were treated with thalidomide and corticosteroid, concomitantly. Of these patients, five suffered a CVE (Table 1).

**Table 1:** Leprosy patients who suffered cardiovascular events in the reported period, during the concomitant use of thalidomide and prednisone.*

Sex	Age	Event	Smoker	SAH	DM	Time of follow-up	Time of TH use
Female	38	Iliofemoral DVT	No	Yes	No	9 m	2 m
Male	45	DVT in R lower limb	Yes	No	No	70 m	10 m
Male	49	Splenic infarction and aortic mural thrombosis	No	No	No	39 m	6 m
Male	54	AMI	Yes	Yes	Yes	56 m	11 m
Male	68	Iliofemoral DVT	Yes	Yes	No	74 m	12 m

* DVT, Deep vein thrombosis; AMI, Acute Myocardial Infarction; SAH, Systemic Arterial Hypertension; DM, Diabetes Mellitus; TH, Thalidomide.

There were three episodes of deep vein thrombosis (DVT) in the lower limbs, one acute myocardial infarction and one splenic infarction with aortic mural thrombosis. All events occurred during the concomitant use of medications (Table 2). No CVE occurred in the group of patients who did not receive the medications concomitantly.

**Table 2:** Characteristics of leprosy patients with or without cardiovascular events during follow-up at the Dermatology Service of Hospital das Clínicas, Faculty of Medicine, Botucatu - UNESP between 2012 and 2021.

Variable	With CVE and the use of TH^a^ n = 5 (%)	Without CVE with the use of TH^a^ n = 27 (%)	Without TH use^a^ n = 57 (%)	Prevalence ratio 95% CI^e^	p
Females^b^	1 (20)	11 (40.7)	16 (28.1)	0.62 (0.10 a 3.69)	0.99
Time of follow-up (months)^c^	56 (39‒56)	48 (33‒86)	34 (17‒55)	‒	0.67
Age at follow-up onset (years)^d^	46.8 ± 10	57 ± 13	54.8 ± 15.1	‒	0.19
Diabetes mellitus^b^	1 (20)	5 (18.5)	11 (19.3)	1.05 (0.17 a 6.40)	0.99
Systemic arterial hypertension^b^	3 (60)	10 (37)	23 (40.4)	1.52 (0.71 a 3.28)	0.39
Smoking^b^	3 (60)	2 (7.4)	15 (26.3)	2.96 (1.29 a 6.81)	0.07
Used TH with CT in the follow-up^a^, ^b^	5 (100)	14 (51.9)	‒	6.46 (3.92 a 10.65)	**<0.01**
Time of TH use (months)^a^, ^c^	10 (6‒10)	13 (3‒36)	‒	‒	0.24
Daily dosage (mg)^c^	100 (100‒100)	100 (100‒100)	‒	‒	0.81

a CVE, Cardiovascular Event; TH, Thalidomide; CT, Corticosteroids. No patient without thalidomide use had CVE during the follow-up.

b Fisher’s exact test.

c Mann-Whitney test. Mean ± standard deviation.

d Student’s *t* test. Median (1^st^ quartile-3^rd^ quartile).

e Prevalence ratio between patients with and without CVE.

Moreover, the high prevalence of smoking among CVE cases is highlighted, but was not statistically significant (p = 0.07). On the other hand, among thalidomide users, smoking was associated with the occurrence of CVE (60% × 7.4%; p = 0.02 – Fisher's exact test).

## Discussion

In the present study, the authors identified a rate of 26.3% (5/19) of CVE and 15.8% (3/19) of DVT in leprosy patients who received thalidomide and prednisone concomitantly. However, considering all thalidomide users, the rates were 15.6% (5/32) and 9.4% (3/32), respectively. It should also be noted that many of these patients were smokers. On the other hand, in multiple myeloma patients receiving thalidomide and dexamethasone, the risk of DVT without prophylaxis was reported to be up to 25%, which is close to the present results.^4^

In recent decades, case reports of thromboembolic vascular events have accumulated during the concomitant use of the two studied medications in leprosy patients. Porto et al. (2019) reported 16 cases of DVT, most of them in men, as in the present sample, so they recommend prophylaxis with AAS 100 mg/day when this drug association is used.^9^ In addition to this report, there have been several similar ones, from different countries.^6^, ^10^, ^11^, ^12^

Sharma et al. evaluated the safety of thalidomide use in 25 patients with different dermatological diseases and observed five cases (20%) of DVT.^13^ The physiopathology of the thromboembolic effects of thalidomide is not fully understood, but in animal studies, reduced bleeding time, as well as reduced thrombin and prothrombin time, and increased serum levels of factor IX and fibrinogen were observed.^14^ On the other hand, antiphospholipid antibodies (APA) are often found in leprosy patients.^15^ A 2018 study observed the presence of APA in six of seven patients with multibacillary leprosy who experienced a thromboembolic event,^16^ and the association of APA persistence with age, bacillary load, and clinical form in leprosy patients has also been reported.^17^ Although most reports of vascular events with thalidomide use are venous, there have been reports of arterial events in patients using thalidomide for multiple myeloma.^18^ Similarly, in a study of patients with multiple myeloma receiving thalidomide, seven thromboembolic events were observed in 23 patients, two of them arterial events.^19^ The present study has limitations because it is monocentric, retrospective and identified a small number of events, but the results are in agreement with the literature, extending the observations from other clinical conditions, such as multiple myeloma.

## Conclusion

Multibacillary leprosy patients treated for a type 2 reaction with thalidomide and corticosteroids are at significant risk of CVE, and attention should be paid to signs suggestive of these events, mainly in patients with additional risk factors for CVE, such as smoking. Prospective studies are needed to confirm these observations, as well as to evaluate the benefit of prophylactic measures for these events in leprosy patients.

## Financial support

None declared.

## Authors' contributions

Melissa de Almeida Corrêa Alfredo: Drafting and editing of the manuscript; effective participation in propaedeutics; literature review; critical review of the manuscript.

Juliano Vilaverde Schmitt: Drafting and editing of the manuscript; effective participation in orientation; effective participation in propaedeutics; literature review; critical review of the manuscript; effective participation in orientation; approval of the final version of the manuscript.

Anna Carolina Miola: Effective participation in data collection; drafting and editing of the manuscript; effective participation in propaedeutics; literature review; critical review of the manuscript; approval of the final version of the manuscript.

Simone de Pádua Milagres: Effective participation in data collection; drafting and editing of the manuscript; effective participation in propaedeutics; literature review; critical review of the manuscript; approval of the final version of the manuscript.

Joel Carlos Lasteria: Critical review of the manuscript; effective participation in orientation; approval of the final version of the manuscript.

## Conflicts of interest

None declared.
